# Electrical and magnetic stimulation separately modulates the extent and direction of neurite outgrowth in an ionically conductive hydrogel

**DOI:** 10.1088/1741-2552/adbb1e

**Published:** 2025-04-02

**Authors:** Katelyn Neuman, Xiaoyu Zhang, Bryan Schellberg, Laura H Lewis, Abigail Koppes, Ryan Koppes

**Affiliations:** 1 Department of Chemical Engineering, Northeastern University, Boston, MA, United States of America; 2 Department of Mechanical and Industrial Engineering, Northeastern University, Boston, MA, United States of America; 3 Department of Biology, Northeastern University, Boston, MA, United States of America; 4 Department of Bioengineering, Northeastern University, Boston, MA, United States of America

**Keywords:** magnetic stimulation, electrical stimulation, hydrogels, ionically conductive, neural regeneration, tissue engineering

## Abstract

*Objective*. The use of conductive materials for aiding peripheral nerve regeneration is a promising method to recapitulate native conductance of nerve tissue and facilitate the delivery of exogeneous stimulation for enhanced recovery. This study systematically investigated the effects of applying electrical (ES) or magnetic stimulation (MS) to neurons within new ionically conductive hydrogels. *Approach*. The material properties of ionically conductive Gel-Amin hydrogels (Gelatin methacryloyl (GelMA) + Choline acrylate) were compared to those of GelMA hydrogels. Neonatal rat dorsal root ganglia (DRG) were encapsulated in both hydrogel formulations, subjected to ES or MS, and evaluated for differences in neuronal extension. Peripheral glia, Schwann cells (SCs), were subjected to the same stimuli and their secretion of various neurotrophic analytes were investigated. *Main results*. Gel-Amin hydrogels are 4x more ionically conductive than GelMA hydrogels. The application of electrical stimulation to the encapsulated cells led to a significant decrease (76%) in DRG outgrowth when encapsulated in GelMA versus the Gel-Amin hydrogel. In contrast, MS led to directional neurite extension in a direction perpendicular to the magnetic field gradient. *Significance*. We present here the first report of a controlled, direct comparison of ES and MS on whole DRG in synthetic materials. The combination of ES and MS decreased total neurite outgrowth but led to more directional growth. Aspects of the material and type of stimuli were noted to reduce several cytokine secretion levels from primary SC cultures. These results highlight the importance of understanding material and biophysical interactions to enhance peripheral nerve regeneration.

## 1. Introduction

Successfully repairing peripheral nerve injuries remains a challenge for researchers and surgeons. These injuries can largely impact patients’ lives through symptoms ranging from loss of sensation and function to pain and paralysis [1]. In severe nerve injuries, damage can span more than 3 cm and often affects the entire nerve tissue. The current gold standard for repairing this type of injury requires harvesting of autologous tissue to replace the damaged nerve structure, proving native glia as well as reducing eliminating tension in the injured nerve to support angiogenesis. Despite improvements in the standard of care and decades of research, less than 50% of patients regain normal function, leaving them with a permanent reduction in functional capacity and chronic pain [1–3]. Current preclinical studies have investigated a wide range of approaches ranging from cell delivery to biomaterial/scaffold design. However, the slow rate of axonal growth, which, in the case of severe injury, leads to months of rehabilitation [4]. Therefore, there is a need for new treatments that accelerate axonal regeneration and restore sensory and motor function following surgical intervention. The use of biophysical stimuli has promise for nerve regeneration, with electrical stimulation (ES) and magnetic stimulation (MS) exhibiting potential to modulate and improve the rate of axon growth [5–7]. On going clinical trials has shown ES has clear clinical success especially in maintaining distal muscle health as well as improving the rate of recovery. However, our limited understanding of the underlying biophysical phenomenon necessitates further *in vitro* investigation. This paper investigates the effect of applying electrical or MS to neurons within ionically conductive hydrogels to better understand how these modalities are impacted by ionically conductive substrates.

Hydrogels hold great potential for nerve repair as materials to fill nerve conduits to aid directional axonal growth from the distal to the proximal nerve stump by not only guiding, but also providing a means to deliver exogenous support cells such as endothelial cells, fibroblasts, and Schwann cells (SCs) which in turn direct axonal growth [8]. Hydrogel structures depend on the crosslinking of constituent polymer side chains, which can be tuned to different applications through the addition of functional domains to influence bulk mechanical properties as well as cell adhesion. The high innate ionic conductance of nerve tissue, which allows for rapid electrical signal transmission between neurons, can be mimicked by incorporating conductive materials into the scaffold [9]. When used in combination with ES, conductive hydrogels facilitate targeted delivery of stimulation to the desired site.

There are clear benefits of utilizing conductive hydrogels in combination with ES to improve nerve regeneration. He *et al* engineered an electrically conductive carbon nanotube-incorporated hybrid hydrogel that prompted significantly more outgrowth in dorsal root ganglia (DRG) with the application of ES compared to no stimulation [10]. Wu *et al* investigated a polypyrrole (PPy) nanoparticle-laden hydrogel and found that PC12 cells exhibited an increase in βIII tubulin and neurofilament expression when subjected to ES [11]. In a similar approach, Poly(3,4-ethylenendeioxythriphene) and polystyrene sulfonate (PEDOT:PSS) were incorporated into a polyethylene glycol diacrylate hydrogel and promoted the expression of various neural differentiation markers in DRG cells when treated with ES [12]. While these results are promising, it remains difficult to translate these materials to the clinic. PPy and PEDOT:PSS can be difficult to work with due to their brittleness and insolubility and may leave behind toxic residue as a result of the fabrication process [13, 14]. Polyaniline also lacks biodegradability and would require a secondary removal surgery [15]. Graphene and other carbon-based materials have been reported to be genotoxic and cytotoxic [16, 17]. Most conductive materials investigated are electro-conductive, meaning they derive their conductance from delocalized electrons that flow through conjugated *π*-bonds. Contrarily, the extracellular matrix (ECM) is innately ionically charged, though weakly ionically conductive, derived mainly from organic polymers such as fibronectin and collagen [18]. Because of this, ionically conductive materials such as those studied here may better mimic the electrical signaling in the body [19, 20].

Recently, the functionalization of choline acrylate (ChoA), an ionic liquid (IL), in a gelatin methacryloyl (GelMA) hydrogel has been demonstrated to increase the conductivity [21–23]. ILs are salt-like, ion-dense materials that are liquid at room temperature with a chemical structure composed of an organic cation and an inorganic/organic anion. Our previous work demonstrated that the combination of GelMA and ChoA or Gel-Amin was able to support neurite outgrowth and glial cell viability *in vitro* [23]. These hydrogels have tunable properties (stiffness, conductivity, degradation rate, etc) as well as good optical transmission for traditional microscopy techniques [22, 23]. To date, this is the first and only evaluation of IL biomaterials for peripheral neuron and glia culture. Exploiting these favorable properties, the current study investigates the impacts of applying ES and MS to neonatal rat DRG and SCs encapsulated in Gel-Amin hydrogels to explore the approach of combining biophysical stimulation with ILs to increase neurite extension.

Endogenous electrical fields (EFs) in the body play a large role in directing embryonic development and injury response [24]. EFs with magnitudes between 10–100 mV mm^−1^ have been observed and recorded in biological tissue [25–28]. Exogenous ES has been reported to influence a variety of cell behaviors, such as cell proliferation, apoptosis and necrosis, stem cell differentiation, and cell migration [29–31]. These changes are hypothesized to be primarily driven by charged ionic movement, which can act on cellular ion channels, membrane proteins, integrins, and organelles [31]. Applying exogenous ES to neurons and glial cells has been demonstrated to modulate axon growth and may improve functional recovery by activating cyclic adenosine monophosphate and the upregulation of regenerative associated genes [6, 7, 32–38], affecting axonal guidance, neuronal survival, and neurite outgrowth [6, 33, 39]. Despite these promising results, clinical adoption of ES has been slow due to some key limitations. First, ES protocols for nerve regeneration vary widely in frequencies, voltages, waveforms, duration of stimulation, and experimental setup. Bertucci *et al* summarize and discuss recent *in vitro* and *in vivo* work and Hasiba-Pappas *et al* summarize and discuss recent clinical studies conducted [40, 41]. Second, the amount of current needed to deliver the desired EF can be substantial and increases as a function of the nerve gap. Particularly when applying direct current (dc), in which electrodes are established as either the anode or cathode, high currents can lead to water electrolysis and heating that damages the surrounding tissue. These irreversible faradaic reactions can be avoided by using an alternating current (ac) in which the electrodes alternate as anodes/cathodes at different frequencies. Thus, ac current is most often used in the clinic, with alternating frequencies between 1–20 Hz [41]. Lastly, ES requires electrodes to be in direct contact with the site of interest, making it difficult to deliver stimulation to patients after their surgery is complete. Recent studies have reported on the effects of delivered EFs varying between 0.5 mV mm^−1^ and 500 mV mm^−1^; many investigations applying 100 mV mm^−1^ report promising results *in vitro* [40]. Nguyen *et al* and Chang *et al* observed increased neurite outgrowth and improved nerve regeneration with an EF of 100 mV mm^−1^ applied in both dc and ac [42, 43]. Increases in SC proliferation and neurotrophic factor production in response to an EF of 100 mV mm^−1^ dc [44, 45]. For this current study, we selected an ac EF of 100 mV mm^−1^ with a frequency of 20 Hz due to the physiological relevance and supporting literature.

In contrast to the state of knowledge of ES application, application of a static magnetic field (SMF) as a modality to improve nerve regeneration is in its infancy [46–48]. SMFs do not provide an associated EF, allowing separation of the SMF and ES effects. Like ES, the utilized magnetic field parameters are often inconsistent, with varying durations of exposure, intensity, and field directions [48]. However, magnetic fields can be applied with permanent magnets without the need for invasive electrodes or a power source. Our recent work with low-magnitude SMFs investigated two different magnetic field configurations and their impact on DRG outgrowth [5, 49]. It was observed that a predominately in-plane magnetic field (14 mT) increased total nerve outgrowth by 63% with a combined in-plane (45 mT) and out-of-plane field gradient (15 T m^−1^) guiding directional axonal growth [5]. These prior results are exciting, and this current work aims to directly compare ES and MS on neuronal outgrowth within a three-dimensional scaffold of two different compositions.

Here, neonatal rat DRG and SCs were encapsulated in ionically conducting GelMA and Gel-Amin hydrogels. Both ES and MS were delivered for 1 h d^−1^ for 3 d, consistent with our previous work with SMFs [5, 49], and effects on total neurite outgrowth and orientation were investigated. To our knowledge, this is the first study to investigate the impacts of pairing an ionically conductive substrate with MS in regenerative medicine. For the DRG, the total outgrowth and directionality of the neurites was analyzed following stimulation. SCs were cultured independently to investigate cytokine secretion and changes in commonly secreted proteins. Our results highlight the importance of limiting current density with ES and indicate MS as a viable option for modulating neuronal outgrowth.

## 2. Materials and methods

### 2.1. Hydrogel synthesis and characterization

GelMA was synthesized according to protocols established previously by us and others [50, 51]. A 4% (w/v) gelatin solution composed of gelatin derived from cold water fish skin (Sigma-Aldrich) in Dulbecco’s phosphate buffer solution (DPBS; Sigma-Aldrich) was homogenized over 1 h at a rate of 450 rpm at 60 °C. 16% (v/v) volume of methacrylic anhydride (Sigma-Aldrich) was added to the gelatin solution with a syringe pump at a rate of 0.5 ml min^−1^. The solution was mixed for 3 h and then diluted with a 3x volume of DPBS (60 °C). The solution was passed through a 0.22 *µ*m filter to remove any unreacted gelatin and poured into membrane tubing (12–14 kDa MWC; Spectrum Laboratories, Inc.). The GelMA solution was dialyzed for a minimum of five days in distilled water (60 °C). The GelMA solution was then frozen for 24 h (−80 °C) and lyophilized for at least 5 d. The resulting soft, white polymer was stored in the dark at −20 °C for use on demand.

The IL, ChoA, was generated according to previous protocols [21–23]. In brief, to synthesize ChoA, acrylic acid (Sigma-Aldrich) was added to choline bicarbonate (Sigma-Aldrich) at a 1:1 mole ratio. The solution was allowed to react at 50 °C for 5 h under vacuum and purified overnight under vacuum at room temperature.

GelMA and ChoA structures were confirmed with proton nuclear magnetic resonance (^1^H NMR; 500 MHz, Varian Inova). For GelMA, the proton spectrum was generated by dissolving 1 mg of GelMA in deuterium oxide (D^2^O; Sigma-Aldrich). For ChoA, 50 *µ*l of ChoA was mixed into D_2_O before reading the proton spectrum.

To synthesize hydrogels, a precursor solution was made composed of a photoinitiator, liquid solvent (either Hank’s balanced salt solution (HBSS; Gibco) for material characterization or cell culture media for *in vitro* experiments), GelMA, and for Gel-Amin hydrogels, ChoA. Any differences in preparation are noted in the following sections. 0.5% (w/v) of lithium phenyl-2,4,6-trimethylbenzoylphosphinate (LAP; Allevi) was added to the liquid solvent and sonicated at 40 kHz with a Branson 2510 Ultrasonic Cleaner for five min to completely dissolve the LAP. GelMA and ChoA were then added to the precursor solution.

In our previous study, two different Gel-Amin formulations 7.5% (w/v) + 2.5% (w/v) and were found to support DRG outgrowth and SC viability over seven days as compared to a 10% GelMA Neuman *et al* [23]. Here, we opted to increase the amount of ChoA to increase the conductivity of the material further with an 8% (w/v) GelMA + 3.5% (v/v) ChoA Gel-Amin hydrogel. The percentage of GelMA was optimized to prevent degradation during the experimental timeline and to match the elastic moduli of the 9.75% (w/v) GelMA hydrogel (supplemental figure 2). The solutions were stored for 1 h at room temperature in the dark to allow all the GelMA to dissolve. Then, the homogeneous precursor solutions were photo-crosslinked with blue light (*λ* = 405 nm, 10 W). The exposure time varied as a function of the hydrogel height (0.25 s of exposure time per *µ*m) [52].

The mechanical properties of GelMA and Gel-Amin hydrogels were analyzed with a TA instruments electroforce 3200 universal mechanical platform according to previous methods [23]. In brief, cylindrical hydrogels (Ø = 8 mm, *H* = 4 mm, *n* = 5, Crosslinking time = 16’ 40”) were fabricated and incubated in HBSS at 37 °C for 2 h prior to testing. A dynamic mechanical analysis (DMA) program (WinTest® 7) was applied in compression with the samples submerged in HBSS during testing. The DMA program applied a sinusoidal frequency sweep between 0.5 and 5 Hz with a mean 10% strain. The software automatically applied a Fourier transform, calculating the difference in phase (*δ*) between the dynamic peak-to-peak force function and the dynamic peak-to-peak displacement amplitude. This was used to calculate the elastic modulus (*E*’; equation (1)) and the viscous modulus (*E*”; equation (2)),

\begin{align*}E{^{^{\prime}}} &amp; = \left( {\frac{{{S_o}}}{{{e_o}}}} \right)*{\text{cos}}\left( \delta \right)\end{align*}

\begin{align*}E{^{^{\prime}}} &amp; = \left( {\frac{{{S_o}}}{{{e_o}}}} \right)*{\text{sin}}\left( \delta \right)\end{align*}

where ${S_o}$ is equal to the stress amplitude and ${e_o}$ is equal to the maximum strain amplitude.

The electrical properties of GelMA and Gel-Amin hydrogels were measured using electrochemical impedance spectroscopy (EIS). For this experiment, deionized water was used as a precursor solvent. Cylindrical hydrogels (Ø = 6 mm, *H* = 7 mm, *n* = 5, Crosslinking time = 29’ 10”) were fabricated and placed between two magnesium stick electrodes (Lincoln® Electric). EIS was recorded between 1 MHz and 100 mHz with a sinusoidal amplitude of ±10 mV. Data were analyzed with EC-Lab® Software. The resultant impedance was fit to an equivalent circuit model to obtain the bulk resistance (imaginary impedance = 0) according to previous work [23]. The calculated bulk resistance was used to calculate the conductivity (*C*) according to the following formulas:

\begin{align*}{\boldsymbol{\rho}} &amp; = \frac{{\boldsymbol{R*A}}}{\boldsymbol{L}}\end{align*}

\begin{align*}{\boldsymbol{C}} &amp; = \frac{\boldsymbol{1}}{\boldsymbol{\rho }}\end{align*}

where *ρ* is the resistivity, *A* is the cross-sectional area, and *L* is the length of the sample.

The rate and degree of hydrogel swelling following crosslinking can significantly influence cell viability. To assess the difference in swelling between our GelMA and Gel-Amin hydrogel formulations, cylindrical hydrogels (Ø = 8 mm, height = 4 mm, *n* = 5) were fabricated. Each hydrogel was weighted and then submerged in HBSS. The samples were incubated at 37 °C and reweighed at different time points (2, 4, 6 and 24 h). The swelling ratio calculated at each time point as the swollen hydrogel weight (${W_{\text{S}}}$) divided by the original hydrogel weight (${W_0}$).

### 2.2. Primary cell isolations

All animal work was approved by Northeastern University’s Institutional Animal Care and Use Committee (NU-IACUC, Protocol #20-0207R) and followed the NIH guide for animal use. Whole DRGs were isolated from neonatal (*p*2) mixed-sexed Sprague Dawley rats according to a previously established protocol [53]. Whole spines were removed and trimmed of excess connective tissue. To reduce variability in DRG size and outgrowth, the spine was reduced to the thoracic spine region. Then, the spine was split medially to expose the spinal cord, which was then removed and discarded. Each DRG was gently lifted with fine point forceps and trimmed of excessive nerve root extensions with a 15-blade scalpel. The DRG were stored at 4 °C in Hibernate-A medium (Gibco) for no more than a week prior to experimentation.

SCs were isolated from the sciatic nerves of neonatal (*p*2) mixed-sexed Sprague Dawley rats via a previously established method [54]. Sciatic nerves were removed and maintained on ice in HBSS prior to plating in six-well plates with sterile tweezers. Each nerve was covered in a drop of basic media (Dulbecco’s modified Eagle medium (DMEM; Gibco) + 10% fetal bovine serum (FBS; Gibco), 50 U ml^−1^ Penicillin/Streptomycin (P/S; Sigma-Alrich)) for 1 h to allow for attachment. After 1 h, 1 ml of media was gently added to each well, avoiding detaching each nerve. After the area surrounding the nerve was confluent with fibroblasts (2–3 d), the nerve was lifted and moved to a fresh well plate. This step was repeated 1x. After the nerve has been moved a second time, only SCs are seen migrating out of the tissue. The media in each well was replaced with fresh basic media supplemented with 10 *µ*m of Cytosine Arabinoside (ARAC; Sigma Alrich), an anti-mitotic, for 72 h to remove any contaminating fibroblasts. To further purify the culture, a cell lysis was performed by targeting cells that express CD90 (Cedarlane Labs, CL005AP) with rabbit complement (Cedarlane Labs, CL3331). Prior to experimentation, cell purity was confirmed to be over 98% with immunocytochemistry (procedure detailed below). A rabbit anti-S100*β* (Invitrogen; BSM-52506R) primary antibody and Alexa Fluor^TM^ 546 goat anti-rabbit (Invitrogen: A11035) secondary antibody were utilized for this experiment.

### 2.3. ES and MS platform and setup

**Figure 1.**
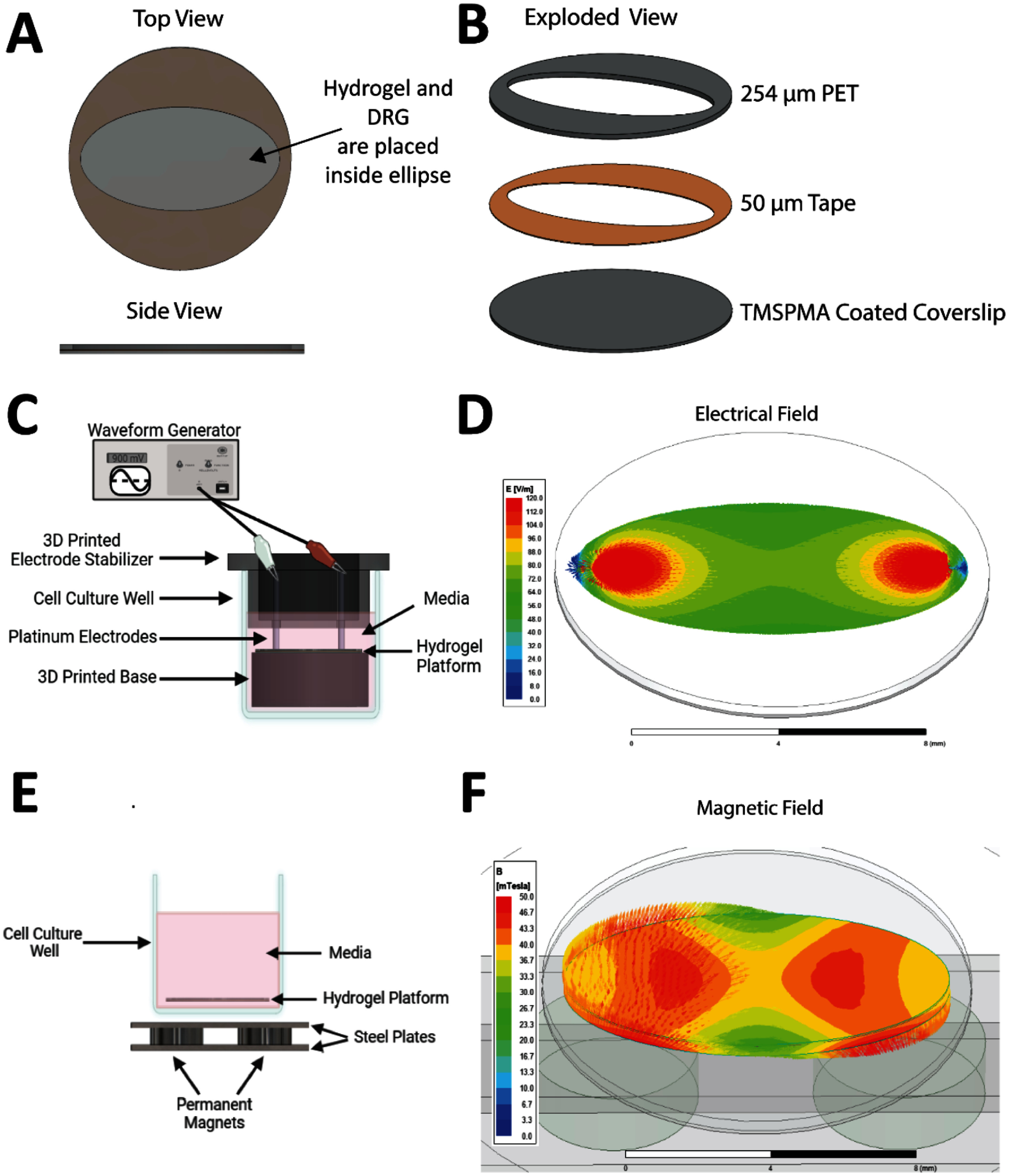
Electrical and magnetic stimulation platform and setup. (A) The top and side view of the laser cut and assembled platform that holds the hydrogel. (B) An exploded view of the platform shows the assembly order of the three different layers: a 254 *µ*m thick layer of PET and a 50 *µ*m thick layer of tape are placed on top of a TMSPMA coated coverslip. (C) Displays the setup for applying electrical stimulation. Created with Biorender.com. (D) ANSYS modeling of the generated electrical field applied. (E) Displays the setup for applying magnetic stimulation. Created with Biorender.com. (F) ANSYS modeling of the generated magnetic field.

Two separate platforms were developed to allow for the application of either electrical or MS in comparable conditions for *in vitro* evaluation (figure 1). For both platforms, the substrate was a 12 mm glass coverslip (VWR) treated with 3-(Trimethoxy silyl) propyl methacrylate (TMSPMA; Sigma Alrich) to improve chemical attachment of the hydrogels when crosslinked. Coverslips were first placed in a bath of 2.8 M NaOH solution overnight at room temperature, and then rinsed thoroughly with distilled water and dipped three times in three separate 100% ethanol baths. The coverslips were allowed to dry completely before wrapping them in aluminum foil and baking at 80 °C for 1 h. The coverslips were then placed in a solution of TMPSMA and baked overnight at 80 °C. The next day, each coverslip was dipped three times in three separate 100% ethanol baths and allowed to dry completely. Lastly, the coverslips were wrapped in aluminum foil and baked for 2 h at 80 °C. Prior to use, the coverslips were stored at room temperature in the dark.

To align DRGs with the applied EF and MF, ellipse shapes were designed as vector images in Adobe Illustrator and cut out of a sheet of 0.01” polyethylene terephthalate PET using an Epilog Zing laser cutter (figures 1(A) and (B)). 3 M Adhesive 966 transfer tape (Ellsworth) was placed on one side of the PET layer prior to laser cutting. The PET and transfer tape were mounted on the TMPSMA coverslips by hand. The platforms were placed in a vacuum at 50 °C for at least 1 week before use to allow the transfer tape to cure and de-gas. Before use for cell culture, the platforms were rinsed three times on a rocker for 1 h with distilled water to remove any contaminates. To sterilize, the platforms were treated with ultraviolet light for 500 s on each side.

#### 2.3.1. ES

A custom-designed apparatus to hold the electrodes and deliver ES was 3D printed (Ultimaker, figure 1(C)). Two parts were designed, and 3D printed to support reproducible application of ES. Each part was designed on SolidWorks and then converted to .gcode. The pieces were extruded with a thermoplastic elastomer (Black PRO Series Flex- 2.85 mm Flexible TPE; MatterHackers) on an Ultimaker 2+. The first design was created with the intent to raise the platform created in the previous paragraph inside a well plate, reducing the height of the electrodes needed to apply ES. The part was a simple cylinder (height = 6.5 mm, Ø = 14.50 mm) with a 12 mm diameter circle indent to keep the platform centered. The second part was modeled off a Transwell to maintain a closed top and two spots for electrodes. The closed top aids in maintaining a sterile environment. The electrode holes were 1 mm in diameter and placed 9 mm apart. Before each use, the 3D-printed pieces were sterilized in 100% ethanol for 10 min and then allowed to dry.

An EF of 100 mV mm^−1^ was generated with a waveform generator (Agilent 33220A, figures 1(C) and (D)). A voltage of ±900 mV was applied as an ac sine wave with a frequency of 20 Hz. This voltage was delivered to the hydrogels via platinum electrodes (Ø = 0.762 mm, Thermo Scientific) connected with electrical wiring to standard alligator clips. The electrodes were in contact with the hydrogel material and circuit completion was checked with a multimeter prior to each stimulation. The electric field profile within the hydrogel at the peak amplitude of the sine wave was modeled using electrostatic simulation in ANSYS Software (Ansys® Electronics Desktop, Maxwell 3D, Release 2020 R1, ANSYS, Inc.), a robust engineering software containing powerful physical models and a built-in library of industry-standardized materials. Simulation confirms that while non-uniform electric field strengths exist near the electrodes, a quasi-uniform in-plane EF of ∼80 ± 10 mV mm^−1^ is present within the cell seeding area—specifically, the central two-mm-radius region, figure 1(D).

#### 2.3.2. MS

MS was delivered using a custom-designed magnetic apparatus, figure 1(E); details of this design are articulated in a previous publication [5]. Briefly, this apparatus employs a pair of neodymium–iron–boron permanent magnets (Ø = 4.8 mm, *t* = 1.6 mm, McMaster-Carr, Grade N52) that were oppositely axially polarized and were sandwiched between two electrical steel pieces (grade M-15, gauge 26 (0.47 mm thickness)) 8 mm apart. This apparatus was placed immediately beneath the culture well to provide a passive SMF to the hydrogel platform. ANSYS magnetostatic simulation indicates a combination of out-of-plane and in-plane field components with an overall field strength of ∼40 ± 5 mT was present within the cell-culturing region, figure 1(F).

### 2.4. DRG encapsulation, culture, and stimulation

GelMA and Gel-Amin precursor solutions were made in a solvent of Neurobasal-A media (Gibco) supplemented with 2 mm L-glutamine (Gibco), 50 U ml^−1^ P/S, 1x B-27 (Gibco), and 25 ng ml^−1^ 2.5 S NGF (Gibco). 27 *µ*l of the hydrogel solution was pipetted into one platform and gently spread with the pipette tip to cover the entire area. A whole DRG was manually placed in the center of the coverslip with tweezers. The hydrogel was photo-crosslinked with blue light for 1 min and 26 s. After crosslinking the platform was placed in a 24-well plate and covered with 250 *µ*l of media. After 15 min, the media was removed and replaced with 500 *µ*l of fresh media to remove any excess photoinitiator. 60 DRG were seeded per trial with ten in each of the six experimental groups (GelMA control, GelMA + ES, GelMA + MS, Gel-Amin control, Gel-Amin + ES, Gel-Amin + MS). DRG were maintained at standard culture conditions (37 °C, 5% CO^2^), 12 h after seeding, the media was fully exchanged and ES and MS were applied to each DRG for 1 h each as detailed in section 2.3. This was repeated the following two days for 3 h total of stimulation per DRG (figure 3(A)). DRG with outgrowth were fixed and imaged as detailed below (*n* = 36, 21, 31, 31, 21, 25). Four independent trials of this experiment were conducted.

### 2.5. Immunocytochemistry

After 7 d of culture, all DRG were fixed with a 4% (v/v) paraformaldehyde (Sigma Alrich) solution for 30 min. The samples were then permeabilized with a 0.1% Triton X-100 (Sigma Alrich) solution for 30 min and blocked with 2.5% (v/v) goat serum (Sigma Alrich) for 4 h. The DRG were incubated overnight at 4 °C with primary antibodies, rabbit anti-S100*β* (Invitrogen: BSM-52506R, 1:250 dilution) and mouse anti-neurofilament-heavy (NF-H; Invitrogen: MA1-2012, 1:200 dilution). The following day, the DRG were washed on a rocker 3x for 1 h each with HBSS. Then, the samples were incubated overnight at 4 °C with 4′,6′-damidino-2-phenylindole (DAPI; Invitrogen: D1306, 1:500 dilution) and secondary antibodies, Alexa Fluor™ 546 goat anti-rabbit (Invitrogen: A11035, 1:500 dilution) and Alexa Fluor™ 647 goat anti-mouse (Invitrogen: A21240). Prior to imaging, the DRG were washed on a rocker 4x for 1 h each with HBSS. Primary and secondary antibodies were diluted in 2.5% goat serum. Imaging was performed with an inverted light microscope (Zeiss Axio Observer, Carl Zeiss Microscopy LLC) at 10x. Images were taken throughout the *z*-direction (*z*-stack) and tiled as needed to capture all neurite outgrowth.

### 2.6. DRG image analysis

DRG neurite growth was assessed quantitatively with ImageJ [55, 56]. For each image, a maximum projection of the *z*-direction in the neurofilament channel was performed prior to analysis. The total outgrowth was measured by manually tracing around the neurite extensions surrounding the DRG. The area of the center DRG ganglion was subtracted from this value. To assess directionally, the radial length of the neurite extensions was measured at 10° angle increments (0°–350°). The angle increments were determined by overlaying polar plot over each maximum projection image. The measured neurite lengths were further binned to represent a major axis (50° ⩽ *θ* ⩽130° and 230° ⩽ *θ* ⩽ 310°) and minor axis (0° ⩽ *θ* ⩽ 40°, 140° ⩽ *θ* ⩽ 220°, and 320° ⩽ *θ* ⩽ 350°). To compare the directional bias between DRG experimental groups, the average neurite length in the major axis was subtracted from the average neurite length in the minor axis. A value of zero for neurite bias represents symmetrical outgrowth, a positive value represents more outgrowth along the major axis, and a negative value represents more outgrowth along the minor axis.

### 2.7. Electrical and MS of SCs in GelMA and Gel-Amin hydrogels

GelMA and Gel-Amin precursor solutions were made in a solvent of SC growth media (DMEM, 10% (v/v) FBS, 50 U ml^−1^ P/S, 10 *µ*g ml^−1^ bovine pituitary extract (BPE; Gibco), 6.6 *µ*m forskolin (Sigma Alrich)). SCs were suspended in each precursor solution at 10 000 cells *µ*l^−1^ density. 27 *µ*l of the hydrogel + cell precursor solutions were pipetted into each platform and gently spread with a pipette tip to fill the entire ellipse. The hydrogel was photo-crosslinked for 1 min and 26 s. The platform was placed in a 24-well plate and covered with 250 *µ*l of media. After 15 min, the media was removed and replaced with 500 *µ*l of fresh media. This was done to remove any excess photoinitiator. Six platforms of SCs were seeded per trial in each of the six experimental groups (GelMA control, GelMA + ES, GelMA + MS, Gel-Amin control, Gel-Amin + ES, Gel-Amin + MS). SCs were maintained at standard culture conditions (37 °C, 5% CO^2^). Full media exchanges were performed on day two and day five. The ES and MS protocols were conserved between DRG and SCs: one hr of stimulation per day, three consecutive days (figure 5(A)). This experiment was repeated three times.

### 2.8. SC analyte secretion

To assess differences in the secretion of various proteins as prompted by the incubation condition, SC media was collected on day two and day five of the experiment, detailed in section 2.8. The supernatants were spun down at 2000 g at 4 °C for 5 min to remove any debris. The supernatant was removed and stored at −80 °C prior to use. A Luminex® discovery assay (Bio-Techne®; LXSARM-07) was performed to detect 7 analytes: interleukin-1*α* (IL-1*α*), interleukin-1β (IL-1*β*), interleukin-6 (IL-6), interleukin-10 (IL-10), tissue inhibitor of metalloproteinase-1 (TIMP-1), tumor necrosis factor-*α* (TNF-*α*), and vascular endothelial growth factor (VEGF). The assay was performed in accordance with the manufacturer’s instructions. Data was collected with a Bio-Plex 200 System (Bio-Rad) using Bio-plex Manager Software (Bio-Rad).

### 2.9. Statistical analysis

Statistical tests were performed with Prism GraphPad. The normality of each data set was assessed with the Shapiro–Wilk test. For parametric data with two experimental groups (mechanical properties, electrical properties, and initial analyte secretion) an unpaired t-test with Welch’s correction was performed. Nonparametric data with two experimental conditions was assessed with a Wilcoxon Rank Sum test (paired nonparametric *t*-test, neurite directionality). For grouped experiments, a two-way ANOVA with a *post hoc* Šidák’s multiple comparison test was used to assess significant differences (analyte secretion overtime). For nonparametric data (total DRG outgrowth), a Kruskal–Wallis test with a *post hoc* Dunn’s multiple comparison test was performed. Error bars on graphs represent the standard deviation. *, **, ***, and **** represent a *p*-value of less than 0.05, 0.01, 0.001, and 0.0001, respectively.

## 3. Results

### 3.1. Materials characterization

#### 3.1.1. Structure

The polymer structure of GelMA was confirmed via ^1^H NMR (supplemental figure 1(A)). Proton peaks at *δ* = 5.6 −5.6 ppm and *δ* = 1.8 ppm, demonstrating the addition of methacryloyl and methacrylamide groups compared to Gelatin. The GelMA spectra also exhibited a reduced free lysine signal (*δ* = 2.9 ppm). The structure of ChoA was also confirmed with ^1^H NMR (supplemental figure 1(B)), with proton peaks at *δ* = 6.1–5.9 ppm and 5.7–5.5 ppm, affirming the acrylation of the choline ion.

#### 3.1.2. Dynamic mechanical properties of GelMA and Gel-Amin hydrogels

**Figure 2.**
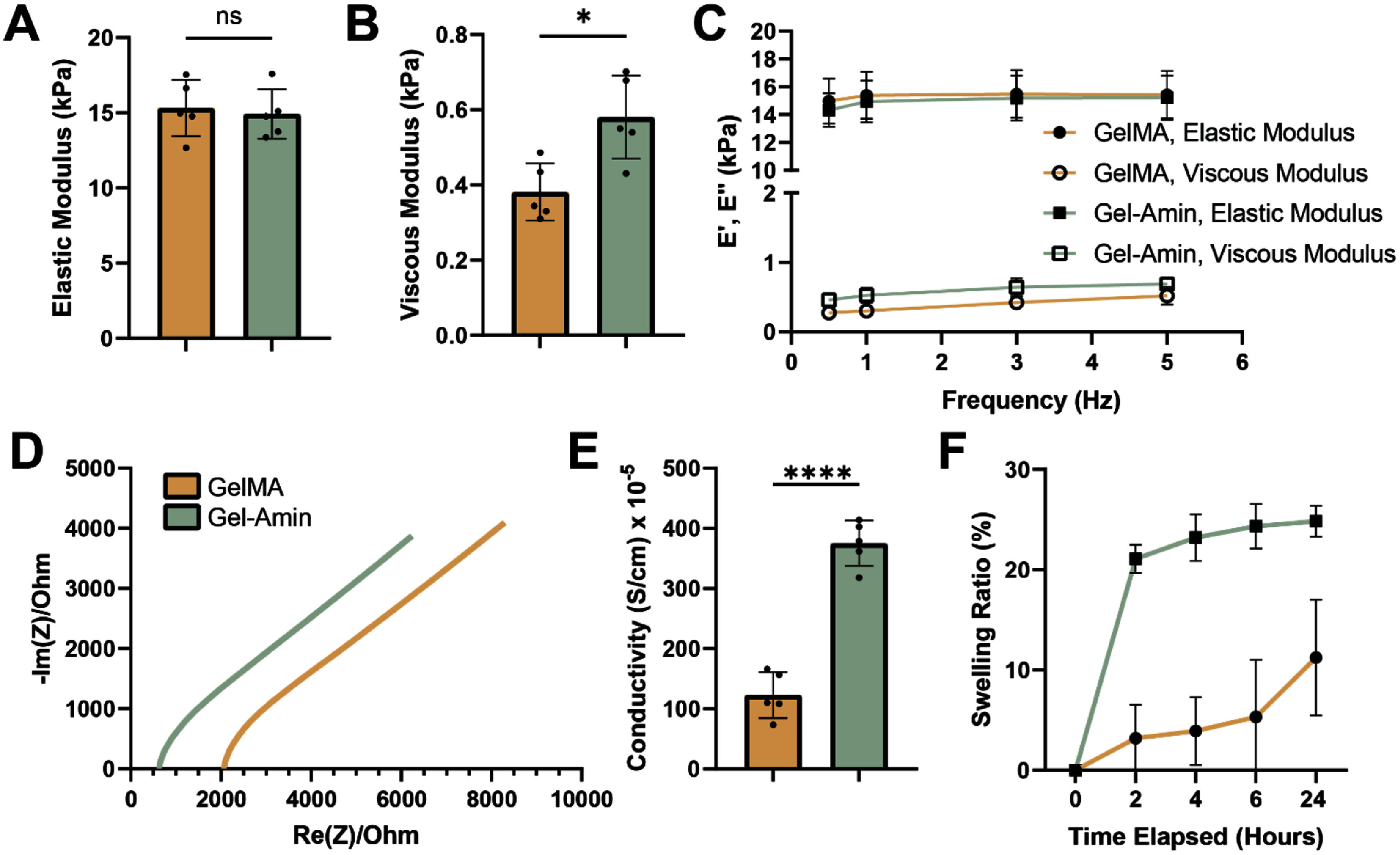
Properties of 9.75% (w/v) GelMA and Gel-Amin (8% (w/v) GelMA + 3.5% (v/v) ChoA) Hydrogels. (A)–(C) Display of the measured mechanical properties: (A) elastic modulus, (B) viscous Modulus, and (C) Elastic and Viscous moduli as a function of frequency. (D) and (E) Display of the measured electrical properties: (D) Nyquist plot and (E) conductivity calculated from the fitted resistance in the Nyquist plot. (F) Display of the swelling ratio as a function of time. Tan and green data trends refer to GelMA and Gel-Amin hydrogels, respectively. Five replicates were measured per experiment. Error bars represent the standard deviations. Data were analyzed with an unpaired t test with Welch’s correction. Significance is indicated by * and **** representing *p* values of <0.05 and <0.0001.

The mechanical properties of various formulations of GelMA and Gel-Amin hydrogels were evaluated to determine compositions of each hydrogel to match elastic moduli (supplemental figure 2). Five different GelMA hydrogel formulations were compared to the selected Gel-Amin hydrogel (8% GelMA + 3.5% ChoA) with an elastic modulus of 14.9 ± 1.7 kPa. The GelMA hydrogels investigated were 9% GelMA, 9.25% GelMA, 9.5% GelMA, 9.75% GelMA, and 10% GelMA. The elastic modulus increased with the increase in GelMA content, ranging from 11.3 ± 1.5 kPa (9% GelMA) to 17.9 ± 1.2 kPa (10% GelMA). The GelMA formulation utilized for all following experiments was 9.75% GelMA. This hydrogel had an elastic modulus of 15.3 ± 1.2 kPa with a *p*-value of 0.73 (unpaired *t*-test with Welch’s correction) compared to that of the Gel-Amin hydrogel formulation (figure 2(A)). The viscous moduli of the GelMA and Gel-Amin hydrogel differed significantly, 0.38 ± 0.08 kPa and 0.58 ± 0.11 kPa, respectively (*p*-value = 0.01, figure 2(B)). The strain frequency or strain rate was found to have minimal impact on the moduli of both hydrogels (figure 2(C)).

#### 3.1.3. Electrochemical properties of GelMA and Gel-Amin hydrogels

The resultant Nyquist plot, figure 2(D), displays the real and imaginary impedances. The bulk resistance (imaginary impedance = 0) was found to be 2068 ± 630 Ω for GelMA and 627 ± 60 Ω for Gel-Amin. Using this resistance, the conductivities were calculated to be 123 ± 38 S cm^−1^ × 10^−5^ and 375 ± 38 S cm^−1^ × 10^−5^, with the Gel-Amin hydrogel being significantly more conductive than the GelMA hydrogel (*p*-value < 0.0001, figure 2(E)).

#### 3.1.4. Swelling properties of GelMA and Gel-Amin hydrogel

GelMA hydrogels gradually swelled over time to 11 ± 5.8% of their original weight (figure 2(F)). The Gel-Amin hydrogels swelled rapidly to 21 ± 1.4% of their original weight after 2 h and then, over time, slowly reached a maximum swelling ratio of 25 ± 1.5%.

### 3.2. Total neurite outgrowth is reduced in GelMA hydrogels with ES

**Figure 3.**
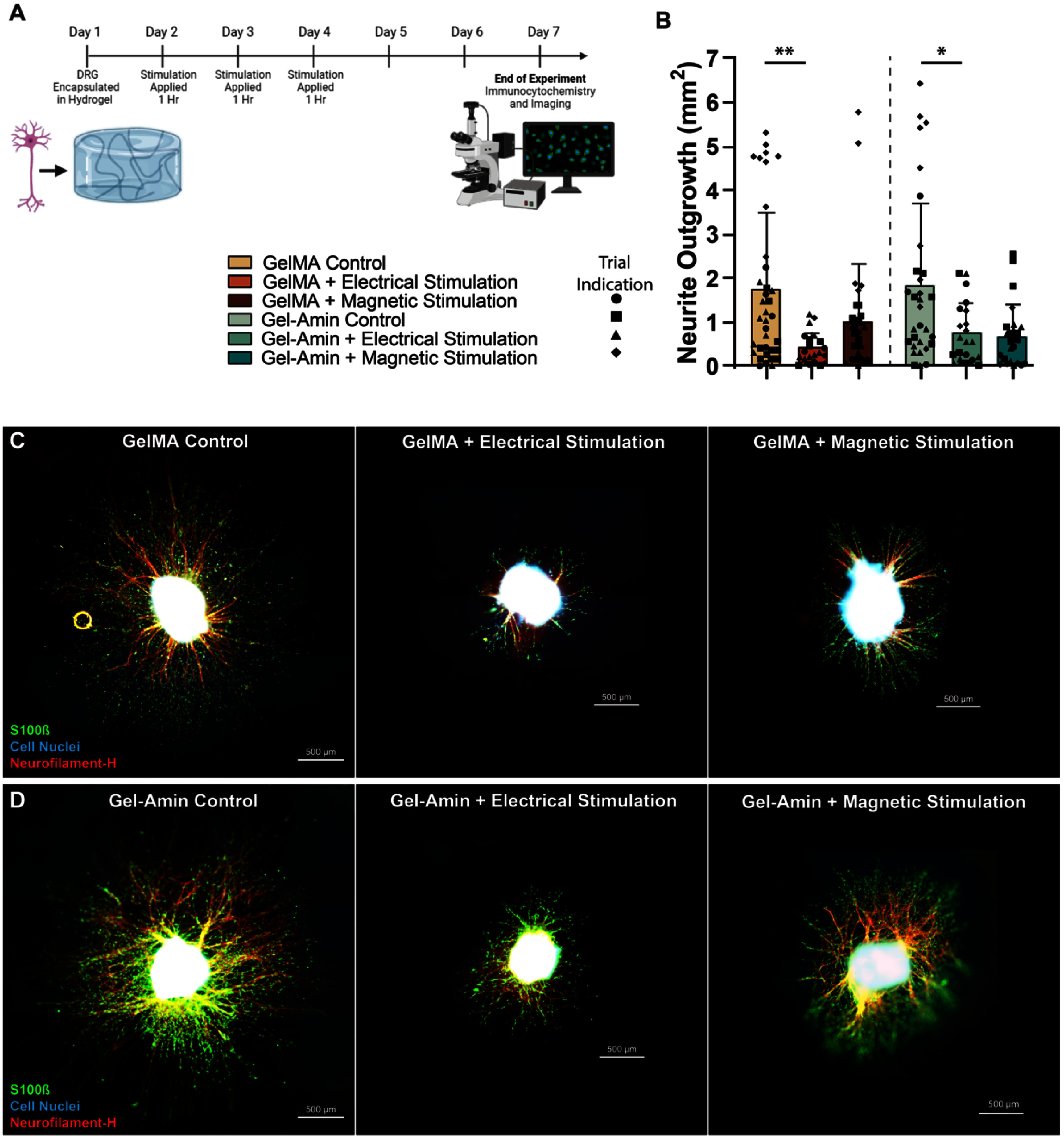
Total neurite outgrowth is reduced in GelMA hydrogels with the application of electrical stimulation. (A) 7 d timeline of the dorsal root ganglia (DRG) experiment. DRG were encapsulated in either a 9.75% (w/v) GelMA or a Gel-Amin (8% (w/v) GelMA + 3.5% (v/v) ChoA) hydrogel and electrical or magnetic stimuli was applied to the respective samples. Created with Biorender.com. (B) Comparison of the total neurite outgrowth area across the six different experimental conditions: GelMA control, GelMA + electrical stimulation, GelMA + magnetic stimulation, Gel-Amin control, Gel-Amin + electrical stimulation, Gel-Amin + magnetic stimulation. Four independent trials were conducted (n = 36, 21, 31, 31, 21, 25) Error bars represent the standard deviation. Data were analyzed with a Kruskal Wallis test with significant differences denoted by * and ** representing *p* values of <0.05 and <0.01. (C) Representative immunofluorescent images of DRG encapsulated in GelMA. (D) Representative immunofluorescent images of DRG encapsulated in Gel-Amin. Green represents S100*β*, red represents neurofilament-heavy, blue represents the cell nuclei (counterstained with DAPI). Scale bars = 500 *µ*m.

After seven days in culture, DRG were processed with immunocytochemistry and imaged to investigate changes in outgrowth under various conditions (figures 3(A), (C) and (D)). Total outgrowth of all DRG was quantified and displayed in figure 3(B). Significant differences in DRG neurite outgrowth were seen between the GelMA control (1.7 ± 1.7 mm^2^) and GelMA + ES group (0.4 ± 0.4 mm^2^), accompanied by a *p-*value of 0.01. There was no significant difference between the GelMA control and the GelMA + MS group (1.3 ± 1.3 mm^2^, *p* = 0.71). The GelMA + ES group also had significantly smaller outgrowth than the Gel-Amin controls (1.8 ± 1.8 mm^2^, *p* = 0.004). There were no significant differences between the Gel-Amin control group and the two stimulation groups, Gel-Amin + ES (0.8 ± 0.7 mm^2^, *p* = 0.57) and Gel-Amin + MS (0.7 ± 0.7 mm^2^, *p* = 0.11). ES of 10–100 mV mm^−1^ did not impact dissociated DRG viability (supplemental figure 3).

### 3.3. MS directs outgrowth along minor axis in GelMA hydrogels

**Figure 4.**
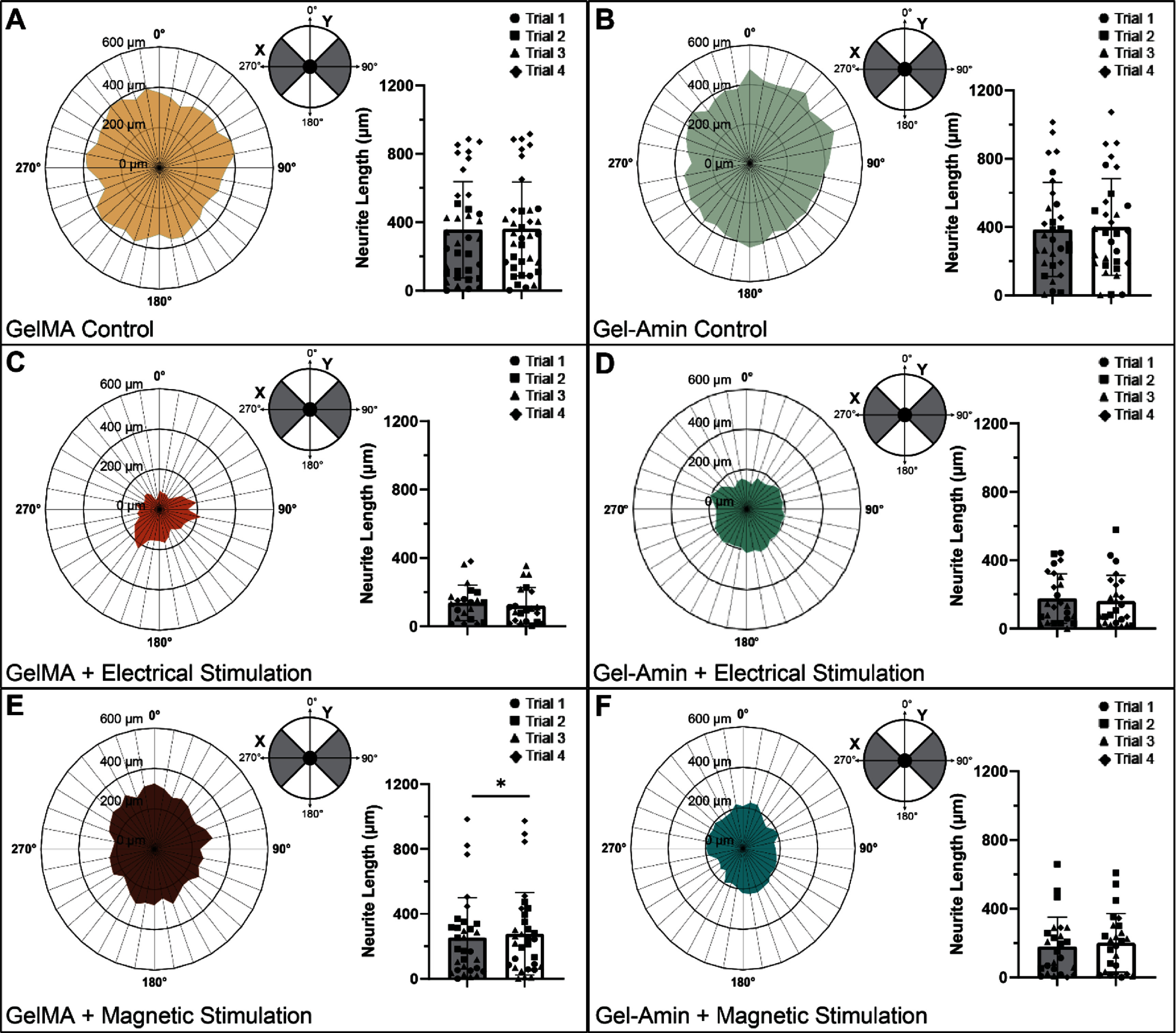
Summary of the directionality of neurite outgrowth in each experimental group. Dorsal root ganglia (DRG) were encapsulated in either a 9.75% (w/v) GelMA or a Gel-Amin (8% (w/v) GelMA + 3.5% (v/v) ChoA) hydrogel and electrical or magnetic stimuli was applied to the respective samples. For each experimental group, the average directionality of neurite outgrowth is depicted with a polar histogram (left) and a direct comparison of neurite growth parallel to the *x*-axis (major axis) and parallel to the *y* axis (minor axis) (right): (A) GelMA Control (*n* = 36), (B) Gel-Amin control (*n* = 31), (C) GelMA + electrical stimulation (*n*= 21), (D) Gel-Amin + electrical stimulation (*n* = 21), (E) GelMA + magnetic stimulation (*n*= 31), and (F) Gel-Amin + magnetic stimulation (*n* = 25). Four independent trials of this experiment were conducted. Significant differences were determined with a Wilcoxon Rank Sum Test (nonparametric paired t-test) and denoted with * representing a *p* value of <0.05. All error bars represent the standard deviation.

The orientation of the neurite outgrowth in each of the six experimental groups was assessed and is shown in figure 4. Four of the groups, both controls and ES conditions, exhibited largely isotropic growth with no significant differences between the major and minor axis (figures 4(A)–(D)). DRG in the GelMA + MS group had a 10% increase in neurite outgrowth along the minor axis (*p* = 0.02, figure 4(E)). While not statistically significant, DRG in the Gel-Amin + MS group also exhibited 10% more neurite outgrowth along the minor axis (*p* = 0.14, figure 4(F)).

### 3.4. Gel-Amin hydrogels reduce SC secretion of IL-1β, TIMP-1, and VEGF

**Figure 5.**
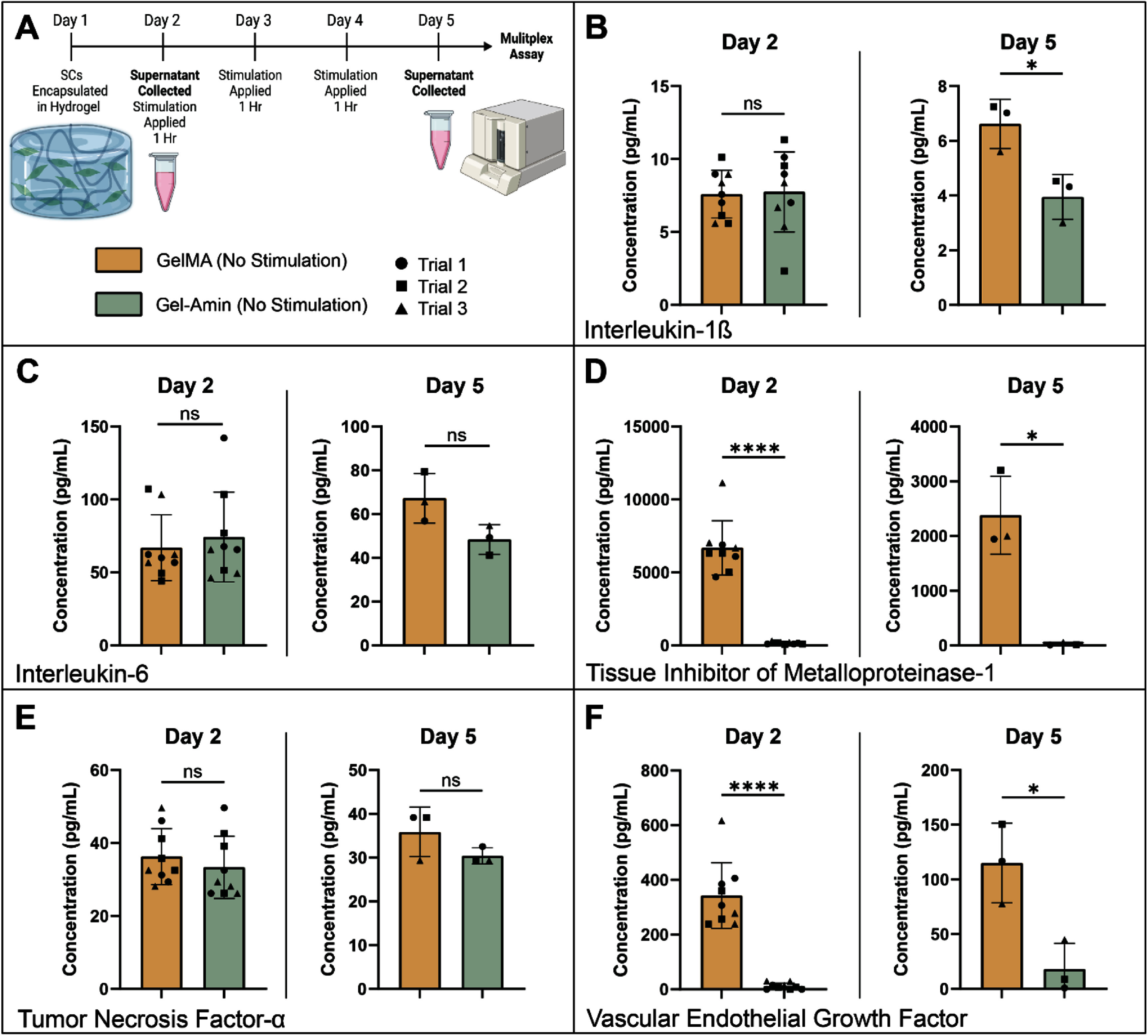
Gel-Amin hydrogels reduce Schwann cell (SCs) secretion of interleukin-1*β*, tissue inhibitor of metalloproteinase-1, and vascular endothelial growth factor. (A) 5 d timeline of the SC experiment with cells encapsulated in either a 9.75% (w/v) GelMA or a Gel-Amin (8% (w/v) GelMA + 3.5% (v/v) ChoA) hydrogel Created with Biorender.com. SC secretion on day two and day five of (B) interleukin-1*β*, (C) interleukin-6, (D) tissue inhibitor of metalloproteinase-1, (E) tumor necrosis factor-α, and (F) vascular endothelial growth factor. Three independent trials were conducted Prior to stimulation all samples per hydrogel were considered replicates (*n* = 9). Error bars represent the standard deviation. Data were analyzed with an unpaired *t* test with Welch’s correction. Significance is denoted by *and **** representing *p* values of <0.05 and <0.0001.

SCs were encapsulated in GelMA and Gel-Amin hydrogels in a similar manner to whole DRG. At day two and day five, media supernatants were collected, and a multiplex bead assay was conducted to assess the secretion of seven different analytes (figure 5(A)). Neither ES or MS exhibited an impact on 2D SC viability (supplemental figure 4). Comparing SCs in GelMA and Gel-Amin hydrogels with no stimulation, there was no difference in IL-1*β* secretion on day two (*p* = 0.89). However, on day five, SCs secreted significantly more IL-1*β* when encapsulated in GelMA hydrogels, 6.6 ± 0.9 pg ml^−1^, compared to that in Gel-Amin hydrogels, 4.0 ± 0.8 pg ml^−1^ (*p* = 0.02, figure 5(B)). There were no significant differences in the secretion of IL-6 at day two (*p* = 0.57) or day five (*p* = 0.07) between the SCs in GelMA and Gel-Amin hydrogels (figure 5(C)). SCs in the GelMA hydrogels secreted significantly more TIMP-1, 6900 ± 1900 pg ml^−1^, on day two (*p* < 0.0001) compared to SCs encapsulated in Gel-Amin, 160 ± 72 pg ml^−1^. This was also true on day five (*p* = 0.03), with SCs in GelMA secreting 2400 ± 710 pg ml^−1^ TIMP-1 while SCs in Gel-Amin only secreted 25 ± 27 pg ml^−1^ (figure 5(D)). There were no significant differences in the secretion of TNF-*α* on day two (*p* = 0.45) or day five (*p* = 0.19, figure 5(E)). On day two, SCs in the GelMA hydrogels secreted significantly more VEGF, 340 ± 120 pg ml^−1^, compared to those in the Gel-Amin hydrogels, 12 ± 11 pg ml^−1^ (*p* < 0.0001). On day five, the SCs in the GelMA hydrogels maintained a higher secretion of VEGF, 120 ± 36 pg ml^−1^, compared to the SCs in the Gel-Amin hydrogel, 18 ± 24 pg ml^−1^ (*p* = 0.02, figure 5(F)). There was minimal secretion of IL-1*α* and IL-10 in SCs in both GelMA and Gel-Amin hydrogels (supplementary figures 5(A) and (B)).

### 3.5. ES and MS impact analyte secretion

**Figure 6.**
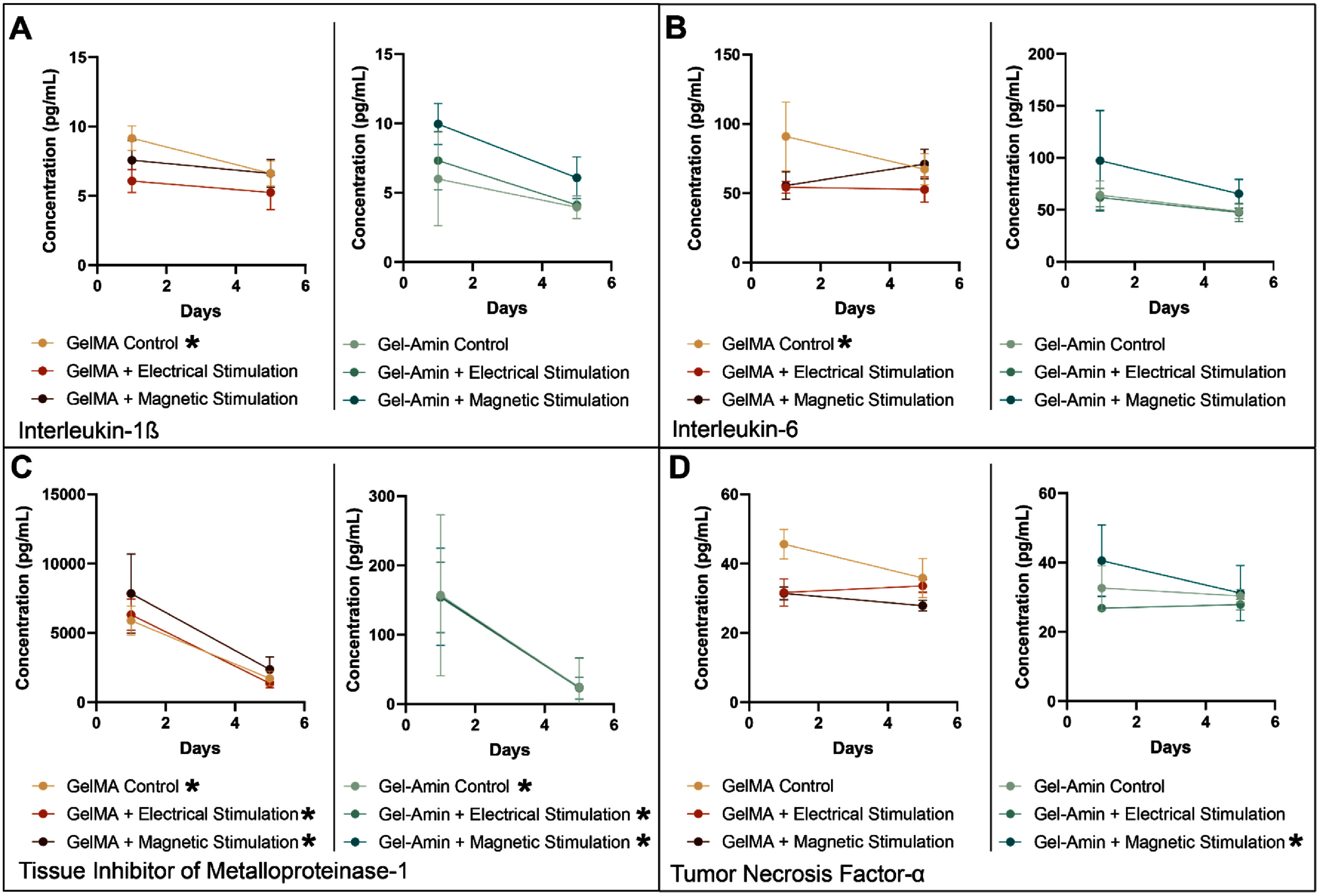
The impact of either electrical or magnetic stimulation on the Schwann cell secretion of (A) interleukin-1*β*, (B) interleukin-6, (C) tissue inhibitor of metalloproteinase-1, and (D) tumor necrosis factor-*α*. Three independent trials were conducted. Prior to stimulation all samples per hydrogel were considered replicates (*n* = 9). Error bars represent the standard deviation. Data were analyzed with a two-way ANOVA with Šidák’s multiple comparison test. Significance is denoted by * in the legend representing a *p* value of <0.05.

Separately, ES and MS were applied to SCs encapsulated in either GelMA or in Gel-Amin hydrogels. Post-stimulation supernatants were collected (day five), and the change over time was paired with their pre-stimulation supernatant (day two) for each analyte (figure 6, supplemental figures 5(C) and (D)). IL-1*β* secretion significantly decreased in the GelMA control from 9.2 ± 0.90 pg ml^−1^ to 6.6 ± 0.90 pg ml^−1^ (*p* = 0.04), while the electrical and MS groups maintained the same secretion level from day two to day five. SCs in the Gel-Amin hydrogels did not change IL-1*β* secretion with the application of the stimulus (figure 6(A)). Similarly, the GelMA control group saw a significant reduction in IL-6 secretion, 91 ± 25 pg ml^−1^ to 67 ± 11 pg ml^−1^ (*p* = 0.03), with the GelMA ES and MS groups maintaining a steady level of IL-6. With the addition of ES and MS in the Gel-Amin hydrogels, SCs did not exhibit any changes in IL-6 release (figure 6(B)). All six experimental groups had significant differences in TIMP-1 secretion over time, with no significant differences seen with the addition of ES and MS (figure 6(C)). There were no differences in TNF-*α* release over time with or without stimulation from SCs in GelMA hydrogels. Applying MS in the Gel-Amin hydrogel led to a significant decrease in TNF-*α* secretion, 41 ± 10 pg ml^−1^ to 31 ± 8.0 pg ml^−1^ (*p* = 0.04). There were no significant differences in TNF-α secretion for the Gel-Amin control and Gel-Amin + ES groups (Figure 6(D)). There were no significant changes in VEGF release over time for any of the six groups (supplemental figures 5(C) and (D)).

## 4. Discussion

In summary, we investigated how an ionically conductive hydrogel, Gel-Amin, influences neuron growth and SC neurotrophin secretion with and without electrical or MS (supplemental figure 6). Information derived from these inquiries is important for informing the design of hydrogel fillers and for understanding how biophysical responses may be altered in this environment. We found that the combination of Gel-Amin and ES was more conducive to neurite growth than GelMA and ES, which led to a significant decrease (76%) in DRG outgrowth (figure 3(B)). While MS did not lead to any changes in total outgrowth compared to the controls, there was a directional bias perpendicular to the magnetic field gradient (figures 4(E) and (F)). This response was seen in both GelMA and Gel-Amin hydrogels, although not significant in the Gel-Amin + MS experimental group. Additionally, we examined the secretion of analytes from SCs after being subjected to the six experimental conditions. Interestingly, the SCs in GelMA hydrogels released significantly more TIMP-1, VEGF, and IL-1β than SCs encapsulated in Gel-Amin hydrogels (figure 5). In the GelMA hydrogels, both ES and MS increased the secretion of IL-1*β* and IL-6 (figures 5(A) and (B)). In the Gel-Amin hydrogels, MS led to a reduction in the secretion of TNF-*α* (figure 6(D)).

Using conductive materials for nerve regeneration is a promising method for recapitulating the native conductance of peripheral nerve tissue and facilitates the delivery of exogenous stimulation for enhanced injury recovery. Unfortunately, there remain limitations on popularly used materials, such as poor processability and cytotoxicity, limiting their translation to the clinic. Incorporating a choline-based IL into tissue-engineered scaffolds offers an alternative to conventional materials. Choline is an essential micronutrient that can be found in a wide variety of food sources, such as eggs, meats, and vegetables and plays an important role in cell growth/division [57]. Previous work demonstrated that ChoA can be incorporated into GelMA hydrogels, dubbed Gel-Amin hydrogels, during crosslinking. The ratio of ChoA:GelMA can be tuned and tailored to various tissue engineering applications [21–23]. In our previous study, two different Gel-Amin formulations (7.5% (w/v) + 2.5% (w/v) and 5% (w/v) GelMA + 5% (v/v) ChoA) were found to support DRG outgrowth and SC viability over seven days as compared to a 10% GelMA [23]. Building off this, we designed Gel-Amin (8% (w/v) GelMA + 3.5% (v/v) ChoA) and GelMA (9.75% (w/v) GelMA) hydrogel formulations further increase conductivity (4-fold increase compared to GelMA) and match their elastic moduli (figure 2). As neurons are well known to be quite sensitive to the mechanical properties of their environment, the hydrogel formulations were optimized to reduce differences that may arise due to differences in substrate stiffness [58–61]. Our Gel-Amin hydrogels exhibit a similar conductance to that of native peripheral nerve tissue (5 × 10^−5^ S cm^−1^) and is comparable to scaffolds fabricated with traditional conductive materials [9, 44, 45].

While encapsulated, DRG in GelMA and Gel-Amin hydrogels exhibited similar total outgrowth (figure 4). To further investigate differences in cell-material interactions, the secretion of seven different analytes by SCs in the two materials was compared (figure 5). After injury, SCs are thought to be the first to sense nerve damage, beginning the process of breaking down and clearing damaged neurites [62, 63]. When the injury site is clear, SCs promote and direct neurite growth [64, 65]. In addition to these important roles, SCs release pro-inflammatory and anti-inflammatory signaling molecules that can recruit other supporting cells [63]. We found that SCs encapsulated in GelMA secreted significantly more IL-1*β*, VEGF, and TIMP-1 than those in Gel-Amin hydrogels (figure 5). The increase in IL-1*β* seen on day five may indicate that the SCs in the GelMA hydrogels are inflamed, as IL-1*β* is a pro-inflammatory cytokine [66]. SCs in the GelMA hydrogels also secreted significantly more VEGF, a growth factor most often secreted in response to low oxygen environments to promote angiogenesis or blood vessel formation *in vivo* [67]. Within the injury site, macrophages excrete VEGF in response to the ischemic environment [8]. VEGF is paramount in angiogenesis and can act as a neuroprotectant *in vivo*. An increase in VEGF *in vitro* may indicate limited oxygen diffusion through the hydrogel material, which could also contribute to the increased IL-1*β* secretion. This hypothesis may be supported by the larger maximum swelling ratio of Gel-Amin (25%) compared to that of GelMA (11%) (figure 2(F)). *In vivo*, forming blood vessels influences SC migration and subsequent Bands of Bunger formation, which relies on VEGF. Further work must evaluate how this decrease in VEGF from SCs translates *in vivo* and impacts macrophages and new blood vessel formation.

Previous work indicated that functionalizing GelMA with ChoA resulted in larger pores [23]. These larger pores in combination with the high salt concentrations, drive rapid and a high degree of swelling into these materials (Figure 2(F)). These observations are consistent with the literature and further discussed in our previous work with this material [23]. These larger pores may also ease cell migration and neurite extension through the polymer network. TIMP-1 was another analyte secreted significantly less in SCs in GelMA hydrogels. This protein is a matrix metalloproteinase (MMP) inhibitor, specifically functioning to inhibit MMP-9 or gelatinase B, an enzyme that plays an important role in ECM remodeling [68]. Other studies have demonstrated that increased amounts of TIMP-1 are linked to decreased cell mobility [69, 70]. These results suggest that Gel-Amin hydrogels may be more conducive to nerve regeneration.

With the application of ES, the DRG in the GelMA hydrogels had significantly less growth compared to both GelMA and Gel-Amin controls. There was also a reduction in total outgrowth in the Gel-Amin hydrogels, though this difference was not as pronounced and was not significantly different than the controls (figure 3). Our previous work has shown that both MS [5, 49] and incorporation of our hydrogel materials [23] do not impact neural cell viability. Still, outcomes reported here may be a result of reduced viability within the DRG not examined here. The morphologies of migrated SCs from whole DRG into the surrounding hydrogel appear viable and healthy. Additionally, while SCs encapsulated in GelMA without any form of stimulation secreted significantly less pro-inflammatory cytokines IL-1*β* and IL-6, when subjected to either electrical or MS, a higher level of the cytokines was maintained (figure 6). While a decrease in neurite outgrowth was not expected, these results confirm that using an ionically conductive hydrogel may be beneficial for nerve regeneration, with future work focused on optimizing stimulation parameters.

Using Ohm’s law, the current applied to the GelMA and Gel-Amin hydrogels to obtain an electric field of 100 mV mm^−1^ was estimated to be 0.44 mA and 1.44 mA, respectively. This is on the high end of currents typically reported in the literature, ranging from 0.5 *µ*A–2 mA [40]. However, it should be noted that more current was used to deliver ES to the cells in the Gel-Amin hydrogel, in which the reduction of DRG outgrowth was not as drastic as the GelMA + ES growth. Perhaps the increase in porosity helped to alleviate the deleterious buildup of reactive oxygen species caused by the high current flux. This points to other variables that may contribute to decreased neurite outgrowth. Multiple stints of stimulation (ex., 1 h a day/multiple days) have not been investigated widely, and studies have conflicting results. Two studies applied repeated ES with a frequency of 20 Hz *in vivo* and saw increased recovery in animals that received stimulation for 1 h a day for 6 d [71, 72]. Contrarily, Park *et al* reported that increased recovery was only seen in animals that received singular ES treatment, and a significant decrease in nerve regeneration was observed when animals received multiple ES (20 Hz) [73]. Al-Majed *et al* applied ES (20 Hz) to the femoral nerve trunk after transection for 1 h, 3 h, 1 d, 7 d and 14 d and found all durations increased motor neuron growth [35]. In a similar study, Geremia *et al* found 1 h was optimal for sensory nerve regeneration and longer time periods decreased axon counts and growth [6]. Our future work will further focus on optimizing the duration of ES with our material formulations.

With MS, the DRG did not experience a difference in total outgrowth; rather, there was a directional change perpendicular to the magnetic field gradient. This 10% difference was significant in DRG encapsulated in GelMA (figure 4). It was still present while not significant in the DRG encapsulated in Gel-Amin. This is particularly intriguing as this directional difference is opposite to what we reported in 2D [5]. This complex system makes it difficult to fully elucidate mechanisms that may be at play. Previously, we hypothesized that the neurites were following the direction of the magnetic field gradient, resulting in more growth parallel to the gradient. As this was not seen when the DRG are encapsulated in a 3D hydrogel, we hypothesized that this change is driven by changes in the hydrogel that occur when exposed to the magnetic field.

In the past, other works have demonstrated the ability of magnetic fields to modify the distribution of electrons and molecules, even modifying conformation changes in amino acid residues and side chains. Due to the symmetry in DRG growth on the left (upward magnetic field) and right (downward magnetic field), we hypothesize that the changes seen are primarily from the Lorentz force perpendicular to the magnetic field. The Lorentz force acts on molecules and ions with electrical charges moving in a magnetic field and can alter local ionic concentrations through natural convection [74, 75]. This could modify the GelMA polymer’s and ChoA’s orientation in a SMF. As DRG and other neurons are highly sensitive to chemokine, voltage, and mechanical gradients, this could explain the perpendicular neurite growth [76–81].

Unique to the SCs encapsulated in the Gel-Amin hydrogels and subjected to MS, we report a significant decrease in TNF-*α* (figure 6). TNF-*α* is an important pro-inflammatory cytokine that mediates immune and inflammatory function [82]. This response aligns with our team’s findings [49], which demonstrated reduced RNA expression of TNF-*α* and its physiological receptors in response to MS in a 2D culture of DRG. This result has also been conserved across recent studies, both *in vivo* and clinically. MS of rats with Parkinson’s disease demonstrated a neuroprotective effect with reduced levels of TNF-*α* as well as cyclooxygenase-2T. Clinically, TNF-*α* serum levels decreased when patients with depression were subjected to repetitive transcranial MS [83]. Here, the significant decrease in TNF-*α* was not as prominent in the SCs encapsulated in GelMA + MS, which may suggest that the ionically conductive material facilitates improved local delivery.

## 5. Conclusions

This work investigated the impacts of biophysical stimulation (electrical/magnetic) in combination with Gel-Amin hydrogels on neurite outgrowth and SC cytokine secretion. This study highlights how substrates can impact the delivery of electrical/magnetic fields and the resultant cellular impacts. Further development and understanding are needed prior to *in vivo* studies where potential variability could arise from differences in implantation technique, tissue composition, and in the directionality of the stimulation. The work presented here will provide the basis to inform future design and development of hydrogel with stimuli combinations for peripheral nerve regeneration, as ionically conductive materials have been demonstrated to alter the expected response.

## Data Availability

All data that support the findings of this study are included within the article (and any supplementary files).
